# Editorial: Personalized care in neurological diseases

**DOI:** 10.3389/fnhum.2023.1216902

**Published:** 2023-05-30

**Authors:** Júlio Belo Fernandes, Catarina Godinho, Tiago F. Outeiro, Cristina Lavareda Baixinho

**Affiliations:** ^1^Escola Superior de Saúde Egas Moniz, Almada, Portugal; ^2^Centro de Investigação Interdisciplinar Egas Moniz (CiiEM), Almada, Portugal; ^3^Grupo de Patologia Médica, Nutrição e Exercício Clínico (PaMNEC), Almada, Portugal; ^4^Department of Experimental Neurodegeneration, Center for Biostructural Imaging of Neurodegeneration, University Medical Center Göttingen, Göttingen, Germany; ^5^Max Planck Institute for Multidisciplinary Sciences, Göttingen, Germany; ^6^Translational and Clinical Research Institute, Faculty of Medical Sciences, Newcastle University, Newcastle upon Tyne, United Kingdom; ^7^Scientific Employee With an Honorary Contract at Deutsches Zentrum für Neurodegenerative Erkrankungen (DZNE), Göttingen, Germany; ^8^Nursing School of Lisbon, Lisbon, Portugal; ^9^Nursing Research, Innovation and Development Centre of Lisbon (CIDNUR), Lisbon, Portugal

**Keywords:** personalized care, neurological diseases, person-centredness, dementia, Parkinson's disease, rehabilitation

## Introduction

Personalized care involves tailoring healthcare services to meet the specific needs of individual patients. This approach recognizes that patients have different circumstances, including age, gender, race, genetics, lifestyle, and environmental factors, which can affect their health outcomes. It also recognizes that individuals have different health needs and experiences. Therefore, a one-size-fits-all approach to care may not be effective (Simmons et al., 2016; Fernandes et al., 2022a). This care approach can involve various activities, including assessing patient needs, developing individualized care plans, and providing appropriate support and resources to help patients achieve their health goals.

Patients with chronic conditions could benefit significantly from a personalized care approach. This can substantially impact their physical and mental health and enhance their ability to self-manage conditions (Fernandes et al., 2022b). In neurological diseases, personalized care can be critical because these conditions can vary widely in respect to symptoms, severity, and response to treatment. By tailoring healthcare services to meet the specific needs of individual patients, personalized care can help enhance patient health outcomes, decrease healthcare costs, and improve the quality of care (Kuipers et al., 2019).

Implementing personalized care requires active participation and dedication from all stakeholders. Policymakers and researchers need to analyze the impact of personalized care on healthcare systems and propose necessary changes to care practices, infrastructures, and policies to fully integrate this care approach (Nardini et al., 2021). Moreover, effective collaboration among patients, caregivers, families, and healthcare providers is crucial for identifying and addressing the challenges related to the patient's condition. In addition, this collaboration helps to develop plans and goals that empower patients and their families.

The Frontiers Research Topic “*Personalized care in neurological diseases*”, featured in the Brain Health and Clinical Neuroscience section of the Frontiers in Human Neuroscience journal, aimed to publish articles describing a personalized care approach to diagnosing and treating patients with neurological diseases. The Research Topic includes four articles.

In the study entitled “Non-game-like training benefits spoken foreign-language processing in children with dyslexia”, Junttila et al. investigated whether plastic changes occur in children with dyslexia more readily after targeted training with a digital language-learning game or similar training without game-like elements. This study's findings indicate that children with dyslexia may benefit more from visually simple training than visually rich game-based training, unlike typical reading children (Junttila et al.).

In the second study, a systematic literature review conducted by Wang et al., entitled “Safety and Efficacy of Cell Transplantation on Improving Motor Symptoms in Patients With Parkinson's Disease: A Meta-Analysis”, identified the efficacy of cell therapy in Parkinson's patients. This review has merits by demonstrating essential clues on the therapeutic effect of cell therapy in alleviating motor impairment and daily living ability in PD patients (Wang et al.).

The third study developed by Avirame et al., entitled “A multimodal approach for the ecological investigation of sustained attention: A pilot study”, aimed to monitor brain engagement by recording ongoing electrophysiological frontal activity. In this pilot study, the researchers investigated a multimodal approach for studying patterns of sustained attention as a function of task modality and complexity. In addition, they also analyzed the brain engagement indices in complex tasks in an exploratory manner to detect outliers and the optimal range of attention. This study's findings support the feasibility of conducting combined electrophysiological and neurocognitive investigations of sustained attention in ecological tasks, providing valuable insights into the patterns of sustained attention based on task modality and complexity (Avirame et al.).

The fourth study consists of the review by Pernes et al., entitled “Documenting falls episodes: a scoping review”, which aimed to map the evidence of the information documenting episodes of falls in older adults. This knowledge is key, as documentation of the fall and the fear of falling, in addition to being a good clinical practice, has the potential to allow health professionals to understand the history of the fall (risk factors, mechanism, and consequences) and develop tailored interventions that prevent its recurrence (Pernes et al.).

As healthcare evolves, developing new care approaches to improve patient outcomes and experiences is vital. One way scientific journals can contribute to this evolution is by developing special issues focusing on new care approaches. In addition, by providing a platform for publishing research studies, reviews, and opinions that explore a particular theme or topic, scientific journals can help advance knowledge, promote new care approaches, and encourage collaboration and cross-disciplinary research. Ultimately, this can contribute to improving patient outcomes and experiences, reduce the burden on healthcare providers, and contribute to the overall advancement of healthcare.

## Author contributions

All authors listed have made a substantial, direct, and intellectual contribution to the work and approved it for publication.
